# Roll-to-roll printing trench-like metasurface film for radiative cooling

**DOI:** 10.1038/s41377-023-01317-w

**Published:** 2023-11-20

**Authors:** Yaoguang Ma

**Affiliations:** 1 State Key Laboratory of Extreme Photonics and Instrumentation, College of Optical Science and Engineering; International Research, Center for Advanced Photonics, Zhejiang University, Hangzhou, 310027 China; 2 ZJU-Hangzhou Global Scientific and Technological Innovation Center, Hangzhou, 311215 China; 3 Jiaxing Key Laboratory of Photonic Sensing & Intelligent Imaging, Intelligent Optics & Photonics Research Center, Jiaxing Research Institute, Zhejiang University, Jiaxing, 314000 China

**Keywords:** Polymers, Metamaterials

## Abstract

A Roll-to-roll technology can enable the fabrication of trench-like photonic meta-structures that are strongly absorptive in the MIR region, providing a controllable optical response for diurnal radiative cooling.

Radiative cooling is a passive process that harvesting the coldness of the universe by transporting the excess heat via thermal radiation. It has been drawing researcher’s attention due to the “free” characteristics since 1960s. But early research has been limited to only nighttime radiative cooling due to the fundamental material challenge which requires the material to have a low absorption in the solar region and a high absorption within the atmospheric window.

The rise of nanophotonic technology provided an efficient way of designing the optical properties of a material at will and offers significantly new opportunities for photon heat flow control. Some inspiring works uses subwavelength structures, including photonic crystals^1^, multilayer thin films^2^, to tailor the radiative photon heat flow of various types of materials. But the practical application of radiative cooling further demands the scalability and cost which forms a tremendous gap between lab-scale demonstrations and real-world systems. The precise control of the spectral properties of scalable radiative cooling materials has been pursued by many researchers, proposing structures like random scatterers^3,4^, random porous polymer networks^5,6^, random synthetic wood^7^, nano-processed silk^8^, etc. But encapsulations with subwavelength glass spheres, titanium dioxide particles or air bubbles will reduce the mechanical strength to some extent. Using a pure polymer to achieve the equivalent effect is always important for real-world scenarios.

Polymer materials are widely used for radiative cooling applications due to the strong absorptive and emissive abilities induced by bond vibrations. The abundant available functional groups vibrating at mid-infrared region, including but not limited to -CHO, C = C, C-O-C, -CF3, etc., make polymer-based radiative cooling materials receive increased attentions. Moreover, the material cost and fabrication cost of polymers are much lower than their inorganic counterparts. These compelling advantages make polymer-based radiative cooling a potential game changer among the other renewable energy technologies.

In the current issue of eLight, joint team from RMIT university, Shandong University and collaborators reported a periodic trench-like metasurface radiative cooling film (PMRC film) fabricated by a roll-to-roll printing technique^9^. The well-designed trench-like structure, as shown in Fig. 1, provides resonance effect that eliminates unwanted reflections in the MIR spectrum region and enhances the absorption of the PMRC film. For a typical film structure, trenches with height D = 2.5 μm, width W = 6.5 μm, and period P = 8 μm were printed on a 50-μm-thick PET film, providing a near unity absorption (96.1%) in the atmospheric window. The back silver coating on the film provides a very high reflectivity (95%) which enables the excellent daytime radiative cooling performance. Furthermore, the trench-structure also exhibits excellent insensitivity to both emitting angle and radiation polarization. The thermal test performed in an isolated testing vehicle further demonstrates over 120 W/m^2^ cooling power under intense solar irradiation over 700 W/m^2^. This PMRC film has also been demonstrated suitable for various cooling scenarios as the polymeric thin film structure has proved ability to adapt to various surface topography. A net cooling power of 80 W/m^2^ has also been demonstrated in a water tank cooling experiment. For both protective clothing cooling and car hood cooling, distinct temperature reduction of more than 10 °C has all been demonstrated.

**Fig. 1:**
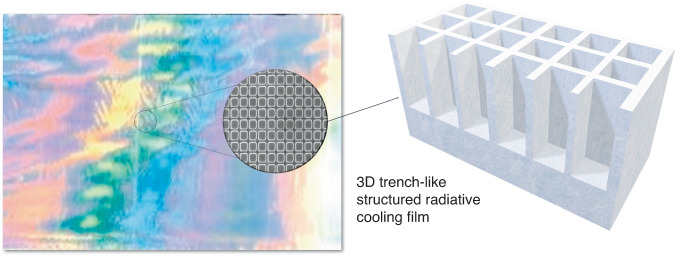
Schematic view of the PMRC film and its microscopic structures

Despite these exciting advances, there is still room for additional efforts. The practical application of radiative cooling technology requires the radiation to be manipulated to adapt different environment conditions. For example, when used in cities, the skyscrapers often block the outgoing radiation and limit the total cooling power^10^. Using this roll-to-roll method, it is possible to fabricate cooling films with subwavelength structures to tailor its radiation directions without losing net cooling power. Also, fabricating microstructures with high scattering coefficients could further spare the metal coating and enhancing the weathering-resistance of PMRC films.

## References

[1] Rephaeli E, Raman A, Fan SH (2013). Ultrabroadband photonic structures to achieve high-performance daytime radiative cooling. Nano Lett..

[2] Raman AP (2014). Passive radiative cooling below ambient air temperature under direct sunlight. Nature.

[3] Zhai Y (2017). Scalable-manufactured randomized glass-polymer hybrid metamaterial for daytime radiative cooling. Science.

[4] Zeng SN (2021). Hierarchical-morphology metafabric for scalable passive daytime radiative cooling. Science.

[5] Mandal J (2018). Hierarchically porous polymer coatings for highly efficient passive daytime radiative cooling. Science.

[6] Li D (2021). Scalable and hierarchically designed polymer film as a selective thermal emitter for high-performance all-day radiative cooling. Nat. Nanotechnol..

[7] Li T (2019). A radiative cooling structural material. Science.

[8] Zhu B (2021). Subambient daytime radiative cooling textile based on nanoprocessed silk. Nat. Nanotechnol..

[9] Lin K (2023). Highly efficient flexible structured metasurface by roll-to-roll printing for diurnal radiative cooling. eLight.

[10] Liu XH (2020). Tunable radiative cooling based on a stretchable selective optical filter. J. Opt. Soc. Am. B.

